# Seven fractions to deliver partial breast irradiation: the toxicity is Low

**DOI:** 10.1186/s13014-017-0825-9

**Published:** 2017-05-23

**Authors:** Marco Trovo, Michele Avanzo, Lorenzo Vinante, Carlo Furlan, Francesco Fiorica, Tiziana Perin, Loredana Militello, Simon Spazzapan, Massimiliano Berretta, Rajesh Jena, Joseph Stancanello, Erica Piccoli, Mario Mileto, Elvia Micheli, Mario Roncadin, Samuele Massarut

**Affiliations:** 1 Department of Radiation Oncology, Centro di Riferimento Oncologico IRCSS, 33081 Aviano, Italy; 2 Division of Medical Physics, Centro di Riferimento Oncologico IRCSS, 33081 Aviano, Italy; 3 Department of Radiation Oncology, University Hospital S. Anna, Ferrara, Italy; 4 Department of Pathology, Centro di Riferimento Oncologico IRCSS, 33081 Aviano, Italy; 5 Department of Medical Oncology, Centro di Riferimento Oncologico IRCSS, 33081 Aviano, Italy; 6 Oncology Centre, Cambridge University Hospitals NHS Foundation Trust, Cambridge, UK; 7 Centro di Riferimento Oncologico IRCSS, 33081 Aviano, Italy; 8 Breast Surgery Unit, Department of Oncology and Surgery, Centro di Riferimento Oncologico IRCSS, 33081 Aviano, Italy; 9 Department of General Surgery, Pordenone General Hospital, 33170 Pordenone, Italy

**Keywords:** Partial breast irradiation, Breast cancer, Toxicity, Fractionation

## Abstract

**Purpose:**

To assess toxicity and clinical outcome, in breast cancer patients treated with external beam partial breast irradiation (PBI) consisting of 35 Gy in 7 daily fractions (5 Gy/fraction).

**Materials and Methods:**

Patients affected by early-stage breast cancer were enrolled in this phase II trial. Patients had to be 60 years old or over and treated with breast conservative surgery for early stage invasive carcinoma.

**Results:**

Seventy-three patients were analyzed. Median follow-up was 40 months. The proposed schedule was well tolerated. No Grade 3 toxicity was documented. Late toxicity was assessable for all the treated patients. Two patients (2.7%) developed Grade 2 pain 6 months after PBI. Four patients (5%) developed asymptomatic fat necrosis. Grade 2 fibrosis was observed in 5 patients (6.7%). No correlation was found between early and late toxicity and the type of adjuvant systemic therapy (no therapy vs. hormonal therapy vs. chemotherapy). No statistical correlation between dosimetric parameters and toxicity was found. Patients who developed Grade 2 radiation fibrosis had not higher radiation volumes to the untreated normal breast than those without fibrosis. Cosmesis was judged good/excellent in the majority of the cases (93%). One patient relapsed locally, and one developed distant metastases, corresponding to a 5-year local control and distant metastases-free survival of 98% and 96.7%, respectively.

**Conclusions:**

35 Gy in 7 daily fractions is an effective and well-tolerated regimen to deliver PBI.

## Introduction

Although whole breast irradiation remains the standard of care after conservative surgery for early stage breast cancer, partial breast irradiation (PBI), namely the irradiation of only the breast tissue surrounding the lumpectomy cavity, is emerging. The recent publication of the American Society for Radiation Oncology (ASTRO) consensus statement gave a further stimulus to the spread of PBI in clinical practice [1].

The most used fractionation scheme is that proposed by Vicini at al., who explored in a phase II trial the safety and efficacy of 38.5 Gy at 3.85 Gy/fraction delivered 2 fractions per day with three dimensional conformal external beam radiotherapy (3D-CRT) [2]. This fractionation was then adopted also by the Radiation Therapy Oncology Group (RTOG): the RTOG 0319 study documented that delivering 38.5 Gy in 5 days, 2 fractions per day, is safe, and the toxicity profile is low [3].

Due to the fact that some studies reported poor cosmetic and toxicity results using the above-mentioned radiotherapy schedule [4, 5], we treated our patients with PBI using a once daily fractionation scheme consisting of 40 Gy in 10 fractions, and we documented an excellent toxicity profile [6]. We therefore decided to continue to accelerate the radiation delivery, according to previously published radiobiological models [7, 8].

The purpose of the present study is to assess prospectively the efficacy and toxicity in breast cancer patients treated who underwent PBI with a schedule consisting of 35 Gy in 7 daily fractions (5 Gy per fraction).

## Materials and Methods

### Patients

Between July 2011 and December 2013 patients affected by early stage breast cancer, who had breast conservative surgery were enrolled in this phase II prospective trial. Both the approval from our institutional review board and written informed consent from the patients were obtained.

To be included in this study patients had to be 60 years old or over and treated with breast conservative surgery for early stage (pT1-T2 pN0-N1a) invasive ductal (IDC) or lobular carcinoma (ILC). Patients were required to have negative surgical margins; re-excision was allowed in case of positive margins. Biopsy of sentinel lymph node was not required. Patients affected by ductal carcinoma in-situ were not included in this study. Adjuvant hormonal therapy or chemotherapy were allowed. In case of chemotherapy, radiation started one month after the completion of it.

### Treatment

**Fig. 1:**
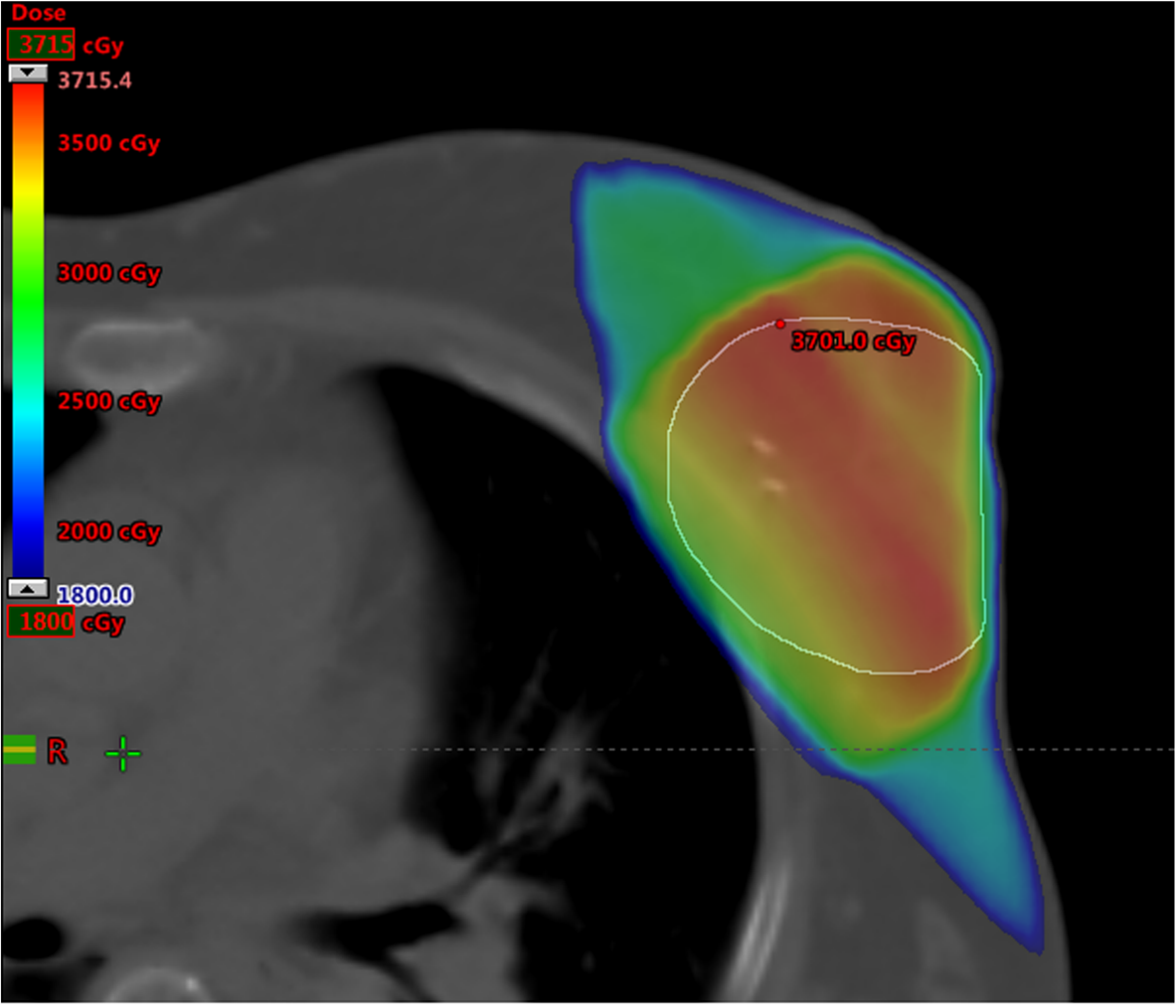
Dose distribution for a partial breast irradiation plan (upper-outer quadrant of the left breast)

Radiation therapy consisted of 35 Gy delivered in 7 daily fractions, 5 Gy/fraction. At least three radio-opaque fiducial markers (surgical clips) were placed in the tumor bed at the time of surgery [9]. Treatment planning for radiation was performed by immobilizing patients in supine position with the Quest Breastboard (Q-Fix System). All patients underwent a complete free breathing computed tomography (CT) simulation to include all the organs at risk (OAR), according to the RTOG 0413 protocol [http://www.rtog.org/members/protocols/0413/0413.pdf]. The CT simulation was performed not before two months following surgery. The clinical target volume (CTV) consisted of the lumpectomy cavity, identified by the surgical clips, uniformly expanded by 10 mm, limited to 5 mm from the skin surface and 5 mm from the lung–chest wall interface. The planning target volume (PTV) was calculated from the CTV using uniform three-dimensional expansion of 5 mm. PTV for evaluation (PTV-EVAL) is the structure used for dose-volume histogram (DVH) constraints and analysis; it is limited to exclude the part outside the ipsilateral breast and the first 5 mm of tissue under the skin and excluding the PTV expansion beyond the posterior extent of breast tissue. Radiation therapy was delivered to the PTV using three dimensional conformal fields, adopting the “field within a field” technique (forward Intensity Modulated Radiation Therapy technique) to improve dose homogeneity within the PTV and dosimetric coverage, as previously described [10]. Dose calculation with tissue inhomogeneity correction was used. All treatments were developed using the Eclipse treatment planning system (Varian Medical Systems, Palo Alto, CA, USA), using multiple planar and non-coplanar 6-MV photon beams. Fig. 1 shows a typical dose distribution. After a treatment plan was approved, digitally reconstructed radiographs (DRRs) were constructed to show each fiducial marker. Radiotherapy treatment commenced 3 to 4 weeks after simulation. The treatment was delivered by a Trilogy linear accelerator equipped with a kV on-board imager system and a 120-leaves Millennium multi-leaf collimator (Varian Medical Systems).

### Follow-up and statistics

Patients were seen at regular intervals to determine the presence of symptoms, and physicians evaluated toxicity by Common Toxicity Criteria of Adverse Events, version 3.0. Follow-up visits for the evaluation of toxicity occurred 1, 3, 6 and 12 months from the completion of PBI for the first year, and then once a year. Cosmesis was assessed using the Harvard scale. Dosimetric parameters from the subgroup with and without Grade 2 or greater radiation induced breast fibrosis were compared using a two-tailed Student’s *t* test; statistical significance was claimed for *p* < 0.05.

## Results

In the study period, 75 patients were enrolled. Two patients were lost at follow-up, therefore the analysis was conducted on 73 patients. Patient and tumor characteristics are reported in Table 1. The median follow-up was of 40 months (range, 7–60 months).

**Table 1:** Patient and tumor characteristics

Age, years	
median	70
range	61–85
Performance Status sec. ECOG	
0–1	66
2–3	7
Side	
Right breast	41
Left breast	32
Histology	
Invasive Ductal Carcinoma	67
Invasive Lobular Carcinoma	6
Tumor dimension, cm	
median	1.2
range	0.5–3.0
T stage	
T1	67
T2	6
N stage	
N0	53
N1mic	8
N1a	2
Nx	10
Estrogen Receptor status	
positive	70
negative	3
Adjuvant therapy	
hormonal therapy alone	47
chemotherapy alone	0
chemotherapy and hormonal therapy	3
none	23

The proposed fractionation regimen was well tolerated. No Grade 3 toxicity was documented. Two (2.7%) patients experience Grade 2 erythema one month after PBI. Four (5.5%) patients developed Grade 1 skin hyperpigmentation. Ten (13.7%) patients had Grade 1 subcutaneous toxicity represented by a mild increased density at palpation of the irradiated tissue.

Late toxicity was assessable for all the treated patients. Two (2.7%) patients developed Grade 2 pain 6 months after PBI. Four (5.5%) patients developed asymptomatic fat necrosis, diagnosed at mammography. Grade 2 fibrosis was observed in 5 patients (6.7%). No cardiovascular events were documented. Cosmetic results were judged “good/excellent” in 69 patients (93%), and “poor” in 6 (7%).

No correlation was found between early and late toxicity and the type of adjuvant systemic therapy (no therapy vs. hormonal therapy vs. chemotherapy). No statistical correlation between dosimetric parameters and toxicity was found (Table 2). In particular, patients who developed Grade 2 radiation fibrosis had not higher radiation volumes to the untreated normal breast (UNB) than those without fibrosis (Table 3).

**Table 2:** Dosimetric data (mean values) of patients with and without Grade 2 radiation fibrosis

	RF (−)	RF (+)	*P* value
PTV_EVAL dimension	119 ml	108 ml	*p* = n.s.
PTV_EVAL/UNB ratio	10.6%	8.7%	*p* = n.s.
PTV_EVAL V95%	97.0%	94.5%	*p* = n.s.
Mean PTV_EVAL Dose	35.2 Gy	35.2 Gy	*p* = n.s.
Heart for Left Located Tumors			
V5%	5.8%	-	
D2 cc (gy)	2.5 Gy		*p* = n.s.
Heart for Right Located Tumors			
V5%	1.2%	4.7%	*p* = n.s.
D2cc (Gy)	1.4 Gy	1.3 Gy	
Ipsilateral Lung			
V15%	18.3%	17.1%	*p* = n.s.
V30%	3.5%	4.2%	
V60%	0.7%	0.9%	
Controlateral Lung			
V3%	less than 1%	less than 1%	*p* = n.s.
Controlateral Breast			
V3%	less than 1%	less than 1%	*p* = n.s.
Thyroid			
V3%	less than 1%	less than 1%	p = n.s.

*Abbreviations: RF* Radiation fibrosis, *UNB* Uninvolved normal breast, *N.s.* not significant

**Table 3:** Uninvolved normal breast dosimetric parameters (mean values) of patients with and without Grade 2 radiation fibrosis

UNB	RF (−)	RF (+)	*P* value
*v5*	75%	80%	*p* = n.s.
*v10*	69%	74%	*p* = n.s.
*v15*	62%	64%	*p* = n.s.
*v20*	54%	55%	*p* = n.s.
*v25*	49%	51%	*p* = n.s.
*v30*	45%	48%	*p* = n.s.
*v35*	42%	45%	*p* = n.s.
*v40*	39%	41%	*p* = n.s.
*v45*	35%	37%	*p* = n.s.
*v50*	32%	33%	*p* = n.s.
*v55*	29%	30%	*p* = n.s.
*v60*	27%	26%	*p* = n.s.
*v65*	25%	23%	*p* = n.s.
*v70*	23%	21%	*p* = n.s.
*v75*	21%	19%	*p* = n.s.
*v80*	19%	17%	*p* = n.s.
*v85*	17%	16%	*p* = n.s.
*v90*	15%	14%	*p* = n.s.
*v95*	12%	11%	*p* = n.s.
*v100*	6%	6%	*p* = n.s.
*v105*	0.2%	0.2%	*p* = n.s.

*Abbreviations: UNB* uninvolved normal breast, *RF* radiation fibrosis, *N.s.* not significant

One patient experienced a local relapse 36 months after the end or radiotherapy, and subsequently underwent radical mastectomy. Local control rate at 5 years was 98%. One patient relapse distantly 3 months after radiotherapy, corresponding to a 5-year distant metastases-free survival rate of 96.7%. This patient died of disease 24 months later; the 5-year overall survival was 98.5%.

## Discussion

In PBI, particularly in the United States, the most commonly used fractionation scheme includes 38.5 Gy delivered in a twice-daily administration. This is based on large, robust and reproducible data, supported both from single institution experiences and from large cooperative groups including the RTOG. Specifically, the RTOG 0319 was a phase II study aiming to evaluate the toxicity of three-dimensional conformal PBI, delivering 10 fractions of 3.85 Gy, twice daily over 5 days [3]. With a median follow-up of 4.5 years only 2 (4%) Grade 3 toxicities were observed. This fractionation and technique were subsequently adopted in the NSABP B39/RTOG 0413 phase III trial, comparing standard whole breast radiation therapy versus partial breast irradiation including the external beam technique [https://www.rtog.org/ClinicalTrials/ProtocolTable/StudyDetails.aspx?study=0413], and also by other large randomized studies [https://clinicaltrials.gov/ct2/show/NCT00282035].

The reason for adopting a one-fraction per day regimen is based on patient preference. A recent study reported survey data describing patient’s breast radiation preferences [11]. Of the 1807 women 70% preferred once-daily radiation therapy for 10 days compared with 30% who preferred the twice-daily option. Interestingly, the multivariate analysis showed that older women were more likely to prefer 10 days of once daily treatment. Moreover, a major factor influencing women’s preference for whole breast irradiation over PBI was likely an aversion to twice-a-day treatment. The authors stated that they believe that their observations support the testing of novel once-daily PBI strategies in clinical trials.

We conducted a phase II prospective study to explore the toxicity profile of a novel fractionation scheme for PBI, consisting of 35 Gy delivered in 7 daily fractions. Using the linear quadratic model and the BED equation derived from this model, assuming an α/β ratio of 4 Gy, as suggested by experiments involving irradiation of human breast cancer cell lines, this prescription would be equivalent to 55.8 Gy in a standard 2-Gy fractionation [12, 13]. These calculations assumed that full repair takes place during the 24-h interval between fractions. In addition, because the hypofractionated regimen also represents an accelerated protocol in which the total dose is delivered in only 7 days, less tumor proliferation is expected to take place compared with that occurring during the standard treatment.

The recent implementation of normal tissue complication probability (NTCP) model for radiation induced fibrosis after PBI supports the hypothesis that this radiation schedule would be well tolerated [7, 8]. According to the model predictions, the present fractionation scheme (35 Gy in 7 fractions) should lead to the same incidence of fibrosis as the previous one, consisting of 40 Gy in 10 daily fractions [6]. Congruent with our hypothesis, we documented that the incidence of Grade 2 fibrosis was of 6.7%. The Grade 2 fibrosis reported with the previous fractionation of 40 Gy in 10 fractions was 5.9% [6].

The low toxicity profile reported in the present study is comparable with the results of other experiences adopting daily fraction schedules for PBI. Investigators at the New York University reported the outcome of 47 patients treated in prone position with 30 Gy at 6 Gy/fraction, delivered in 5 fractions within 10 days; with a median follow-up of 18 months they reported only late Grade 1 toxicity [14]. Researchers from the University of Florence recently published their institutional phase III randomized trial, comparing whole breast irradiation vs. external beam PBI consisting of 30 Gy in 5 daily fractions [15]. The PBI group presented significantly better results considering acute and late toxicity, and no Grade 2 or higher toxicities were observed in this group.

## Conclusions

In conclusion, we showed that a 7 fractions schedule for PBI is safe, although a longer follow-up is needed to further ascertain late toxicity, and in particular late fibrosis. Based on the data reported in the present paper, we will continue our research in the direction of reducing the number of fractions for external beam PBI. We are now enrolling patients in a phase II trial designed to deliver 28 Gy in only 4 daily fractions.

## Abbreviations

3D-CRT: Three dimensional conformal external beam radiotherapy
ASTRO: American society for radiation oncology
CT: Computed tomography
CTV: Clinical target volume
DRRs: Digitally reconstructed radiographs
DVH: Dose-volume histogram
IDC: Invasive ductal carcinoma
ILC: Invasive lobular carcinoma
PBI: Partial breast irradiation
PTV: Planning target volume
PTV-EVAL: Planning target volume for evaluation
RTOG: Radiation therapy oncology group
UNB: Untreated normal breast
